# Adipocytes secreted leptin is a pro-tumor factor for survival of multiple myeloma under chemotherapy

**DOI:** 10.18632/oncotarget.13342

**Published:** 2016-11-14

**Authors:** Wen Yu, De-Dong Cao, Qiu-bai Li, Hui-ling Mei, Yu Hu, Tao Guo

**Affiliations:** ^1^ Department of Hematology, Union Hospital, Tongji Medical College, Huazhong University of Science and Technology, Wuhan, Hubei, China; ^2^ Department of Pediatrics, Tongji Hospital, Tongji Medical College, Huazhong University of Science and Technology, Wuhan, China; ^3^ Department of Oncology, Remmin Hospital of Wuhan University, Wuhan, Hubei, China; ^4^ Collaborative Innovation Center of Hematology, Huazhong University of Science and Technology, Wuhan, Hubei, China

**Keywords:** leptin, adipokines, multiple myeloma, chemotherapy

## Abstract

Accumulating evidences have shown that adipokines secreted from adipocytes contributes to tumor development, especially leptin. However, underlying mechanisms remain unclear. This study aims to explore the effect of leptin on development and chemoresistance in multiple myeloma cells and the potential mechanism. Analysis of levels of adipokines including leptin and adiponectin in 28 multiple myeloma patients identified significantly higher leptin compared with 28 normal controls(*P* < 0.05), and leptin level was positively correlated with clinical stage, IgG, ER, and ß2MG. Next, by using co-culture system of myeloma and adipocytes, and pharmacologic enhancement of leptin, we found that increased growth of myeloma cells and reduced toxicity of bortezomib were best observed at 50 ng/ml of leptin, along with increased expression of cyclinD1, Bcl-2 and decreased caspase-3 expression. We also found that phosphorylated AKT and STAT3 but not the proteins expression reached peak after 1h and 6h treatment of leptin, respectively. By using AG490, an agent blocking the phosphorylation of AKT and ERK, the proliferation of myeloma cells was inhibited, as well as the phosphorylation of AKT and STAT3, even adding leptin. Taken together, our study demonstrated that up-regulated leptin could stimulate proliferation of myeloma and reduce the anti-tumor effect of chemotherapy possibly via activating AKT and STAT3 pathways, and leptin might be one of the potential therapeutic targets for treating myeloma.

## INTRODUCTION

Multiple myeloma(MM) is an incurable malignancy of plasma cells that accumulate in the bone marrow. MM strike males and females in elders and represent the second most common hematological cancer [1]. It is characterized by excessive production of heavy and light chain monoclonal immunoglobulin, as well as bone destruction [2, 3]. Currently there are several established risk factors: older age, meaning unknown monoclonal immunoglobulin hyperlipidemia (MGUS) and family history of lymphatic cancers [4, 5]. Some potential risk factors such as exposure to chemicals and radiation, chronic immune activation, are still under determination [6–8]. In recent years, more and more evidence show that obesity is a risk factor for the occurrence of MM [8]. As obesity accompanied with increased adipose tissue, that is not only an organ for energy savings, but also an endocrine organ that secretes adipokines. The adipokines takes part both in the metabolism, fat synthesis and degradation and regulation of human inflammatory response [9, 10]. One reason for obesity increase the risk of developing multiple myeloma may be associated with changes in adipokines levels.

Adipocytes are differentiated from mesenchymal stem cells and a major type of bone marrow stromal cells(BMSCs). Adipocytes are the primary components of adipose tissue and secret several adipokines. A number of researches have proved that changed levels of the adipokines secreted from adipose tissue is thought to be involved in the development of several types of cancer [8, 10, 11]. Leptin and adiponectin are two members of the adipokines and well-studies in association with tumors. In recent years, studies revealed that levels of leptin and adiponectin were changed in MM patients and other cancer [9, 10, 12–14].

Leptin is important for energy balance and metabolism, as well as adaptive immune function, receptors associated angiogenesis and hematopoietic process [14]. As reported, in fatty population with increased risk of cancer, the level of leptin usually significantly elevated, suggesting that leptin may also be involved in cancer development [15–17]. Previous studies also suggested that it protected cancer cells from chemotherapy agents induced apoptosis *in vitro* by reducing intracellular reactive oxygen species(ROS) [18]. Adiponectin is another member of adipokines secreted by fatty cells with hormone characteristics. Previous studies showed an inverse correlation between plasma concentration of adiponectin and incidence of breast, prostate, colorectal, and acute myeloid leukemia [9, 10, 13, 19]. Adiponectin is suggested to be a protective factor by exerting anti-inflammation, insulin-sensitizing, and anti-angiogenesis [20–22].

Although previous studies showed that certain kinds of adipokines were related to risk of myeloma, the characteristics of correlation and mechanism remain unclear. Therefore, in this study, we evaluated the levels of adipokines (leptin and adiponectin), determining the correlation between their disturbances and clinical characteristics in multiple myeloma, and investigated the possible underlying mechanisms between adipokines and multiple myeloma, and whether aberrant expression of adipokines could be served as a novel target for preventing myeloma chemo-resistance.

## RESULTS

### Adipokines levels in the serum of patients with multiple myeloma

28 patients with multiple myeloma and 28 control cases were allocated to measure serum adipokines levels. Distribution of study factors is presented in Table 1. BMI, which is associated with MM, was slightly lower among selected MM patients than among controls. There were 15 male and 13 female in the MM group, as well as the control group. The mean age of MM patients were 56 years, and it was 55 years in the control group. There were no significant difference among all of these baseline characteristics.

**Table 1:** selected characteristics of MM patients and matched controls

	MM	control
cases	28	28
M, n/F, n	15/13	15/13
age, mean (SD)	56 ± 10	55 ± 12
BMI, mean (SD)	22.79 ± 3.1	23.01 ±2.8
MM ISS stage, n (%)		
I	5 (17.9%)
II	7 (25%)
III	16 (57.1%)

Abbreviations: MM, multiple myeloma; M, male; F, female; BMI, body mass index; SD, standard deviation.

### Levels of adipocytokines in MM and control

**Figure 1:**
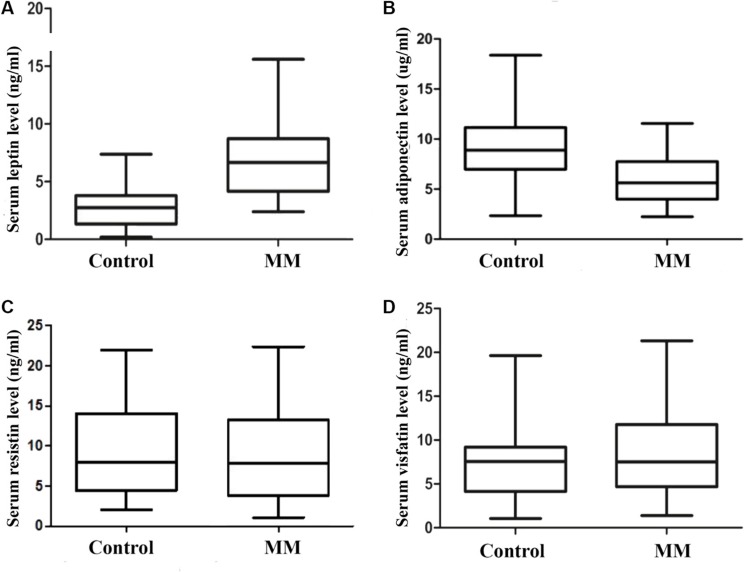
Serum adipocytokines levels in control group(*n* = 28) and MM group(*n* = 28) The level of leptin is significantly higher in MM group when compared with control group. (**A**) serum leptin level; (**B**) serum adiponectin level; (**C**) serum resistin level; (**D**) serum visfatin level).

By using ELISA, we measured serum levels of adipocytokines, including leptin, adiponectin, resistin, and visfatin. As shown in Figure 1A, MM patients had higher levels of leptin than did controls (6.82 ± 3.09 vs. 2.91 ± 1.81; *p* <0.01). The level of adiponectin in MM patients was significantly lower than control group(5.79 ± 2.37 vs. 9.29 ± 3.45; *p* < 0.01. Figure 1B). However, there were no significant differences in levels of resistin(8.98 + 6.41 vs. 9.48 ± 6.18, *p* = 0.091. Figure 1C) and visfatin(8.35 + 5.06 vs. 7.74 ± 4.79 ng/ml, *p* = 0.819. Figure 1D) between MM and control groups (Table 2).

**Table 2:** Serum adipocytokines levels in control group and MM patients

Mean + SD	MM patients *N* = 28	control *N* = 28	*P* value
Leptin (ng/ml)	6.82 ± 3.09	2.91 ± 1.81	< 0.01
adiponectin (μg/ml)	5.79 ± 2.37	9.29 ± 3.45	< 0.01
resistin (ng/ml)	8.98 ± 6.41	9.48 ± 6.18	0.091
visfatin (ng/ml)	8.35 ± 5.06	7.74 ± 4.79	0.819

Abbreviations: MM, multiple myeloma; SD, standard deviation.

### Correlation between serum levels of leptin and adiponectin and clinical features

As shown in Tables 3 and 4, we observed inverse associations between MM clinical features and plasma levels of leptin and adiponectin. Consistent patterns of association were observed for leptin (*p* = 0.023) and adiponectin (*p* = 0.015), when the MM ISS stage was subdivided using the Stage I + II vs. Stage III.

**Table 3:** Correlation between serum leptin and adiponectin levels and clinical parameters in MM patients

	MM ISS stage	Mean	SD	*P* value
leptin	Stage I + II	5.34	1.9	0.023
adiponectin	Stage I + II	7.05	2.62	0.015

Abbreviations: MM, multiple myeloma; SD, standard deviation.

**Table 4:** Correlation between serum leptin and adiponectin levels and clinical parameters in MM patients

	leptin		adiponectin	
	Spearman r	*P* value	Spearman r	*P* value
IgG	0.607	0.001	–0.575	0.001
MM cell %	0.513	0.005	–0.065	0.741
ß2 MG	0.572	0.01	–0.577	0.001
ESR	0.374	0.05	–0.441	0.019
Serum albumin	0.546	0.003	0.517	0.005
LDH	–0.014	0.943	–0.179	0.362
Hb	0.279	0.151	0.262	0.179
serum calcium	–0.275	0.156	0.264	0.175

Abbreviations: MM, multiple myeloma; MG, microglobulin; ESR, Erythrocyte Sedimentation Rate; LDH, lactate dehydrogenase.

Pearson coefficient correlation showed a significant positive relation between leptin and IgG level, ER, bone marrow plasma cells % as well as ß2MG, but not the LDH, hemoglobin or Ca2+. Adiponetin level was inversely correlated with IgG level, ß2M and ER. Whereas, there were no significant differences in resistin level and visfatin level between MM patients and healthy individuals. Also we haven’t been able to find either a significant association between resistin level and MM prognostic biological parameters or a significant association between visfatin level and MM prognostic biological parameters (Table 4).

### Generation of human adipocytes *in vitro*

We have found adipokines levels were associated with clinical characteristics in multiple myeloma patients. Because bone marrow cavity is filled with adipocytes and their numbers is strongly correlated with age, in this part, we investigate the effect of adipocytes on the biological behavior of multiple myeloma.

**Figure 2:**
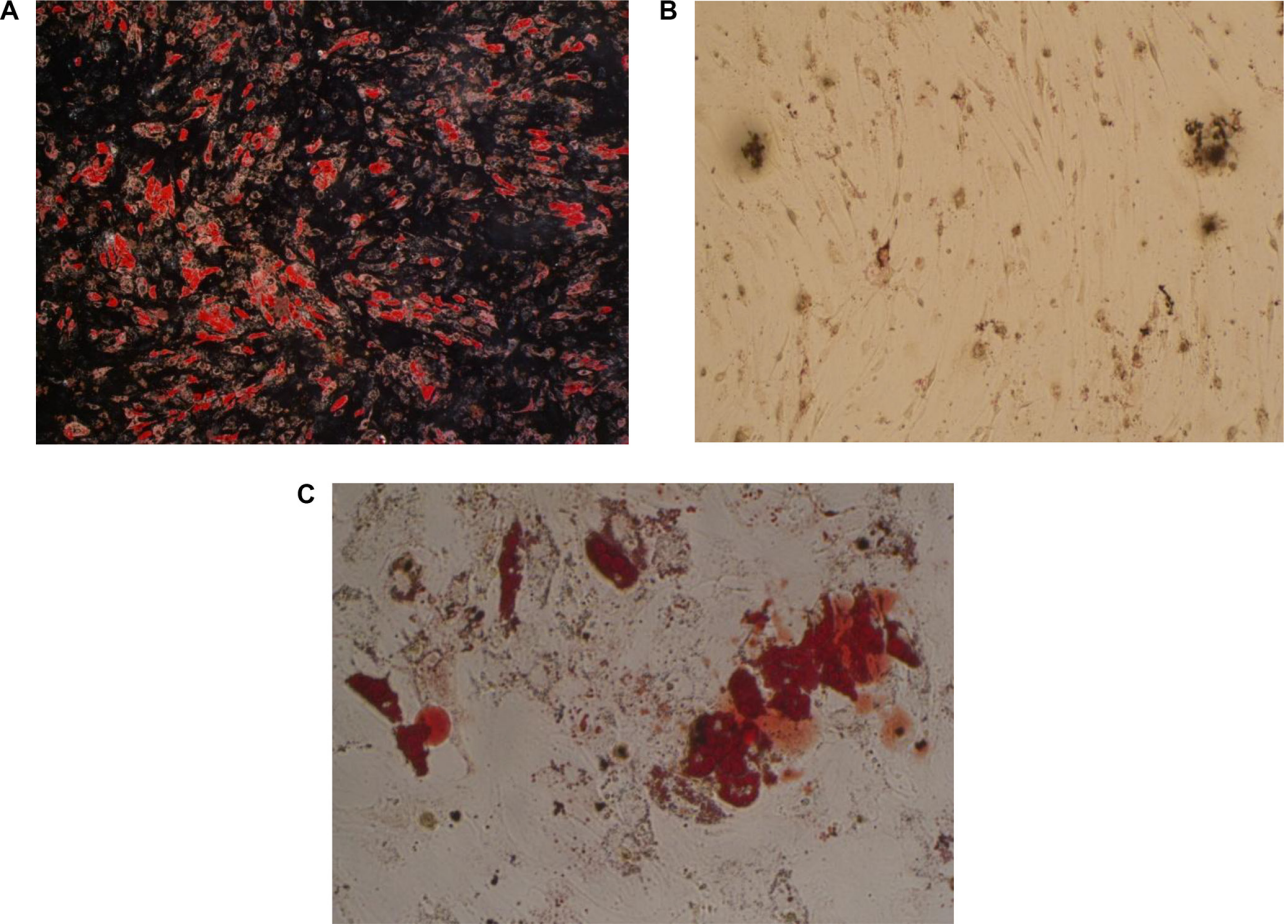
Detection of human adipocytes *in vitro* by oil red statin (**A**) mature fat cells differentiated from 3T3-L1 cells; (**B**) undifferentiated mesenchymal stem cells; (**C**) mature fat cells differentiated from mesenchymal stem cells).

3T3-L1 cells and isolated human MSCs from iliac crest bone marrow aspirates of 6 healthy volunteers were later differentiated into adipocytes by culturing them in adipocyte medium for nearly 2 weeks. During the adipocyte-differentiation culture period, more and more 3T3-L1 cells or MSCs showed an accumulation of multiple lipid droplets. 80%–90% of the 3T3-L1 cells were differentiated into adipocytes while 20%–30% of the MSCs were differentiated into adipocytes. Both of the two kinds of mature adipocytes were detected with Oil Red stain (Figure 2). The generated mature adipocytes were further purified for the following experiments.

### Level of leptin released by the 3T3-L1 and MSCs generated mature adipocytes

The concentrations of leptin in the culture supernatant of both adipocytes were tested by ELISA. The levels of leptin in 3T3-L1 cells and MSCs differentiated adipocytes were 0.88 ± 0.17 ng/ml and 1.52 ± 0.12 ng/ml, respectively.

### Adipocytes promotes multiple myeloma cells proliferation via leptin and its receptor

To investigate whether adipocytes have a beneficial role in myeloma cell proliferation, we cultured myeloma cells including RPMI8226, ARH-77, U266, NCI-H929 with or without adipocytes for three days. The proliferation rates(PR) were tested by CFSE staining. The results showed that the proliferation rates were significantly higher in co-culture systems, compared with control. The PRs for ARH, NCI-H929, RPMI8226 and U266 co-culture cells were 1.28 ± 0.07, 2.19 ± 0.18, 1.26 ± 0.09, and 1.48 ± 0.07, respectively. These data indicate that adipocytes stimulating proliferation in myeloma cells.

**Figure 3:**
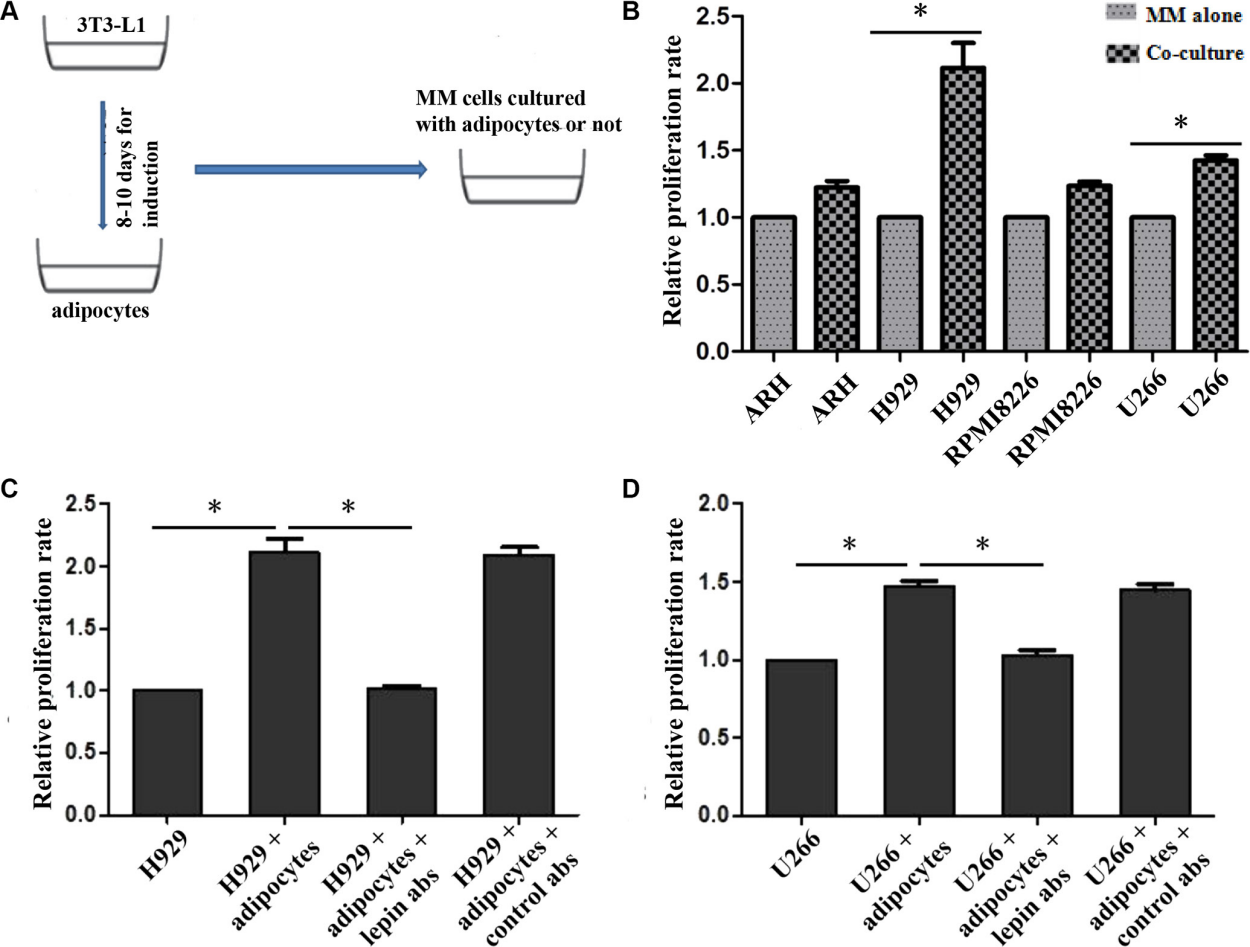
Proliferation of MM cells when co-cultured with adipocytes The proliferations of MM cells are increased when co-cultured with adipocytes, and H929 and U266 are most significant (**B**). However, this pro-proliferation effect is significantly inhibited after adding leptin receptor antibodies both in H929 (**C**) and U266 cells (**D**). (**A**) overall process of co-culture system; B, relative proliferation rates of ARH, H929, RPMI8226, and U266 cells(*n* ≥ 3); C and D, proliferation rates of H929 and U266 were impaired when adding leptin receptor antibodies to the culture systems. *p* <0.05).

As leptin level was significantly increased in myeloma patients, we tried to determine whether the pro-proliferation effect of adipocytes was through leptin or not. Anti-leptin receptor antibody was administrated in co-culture system of NCI-H929 + adipocytes and U266 + adipocytes, and the growth of myeloma cells was suppressed (Figure 3).

### Adipocytes protects multiple myeloma cells from apoptosis induced by chemotherapy

**Figure 4:**
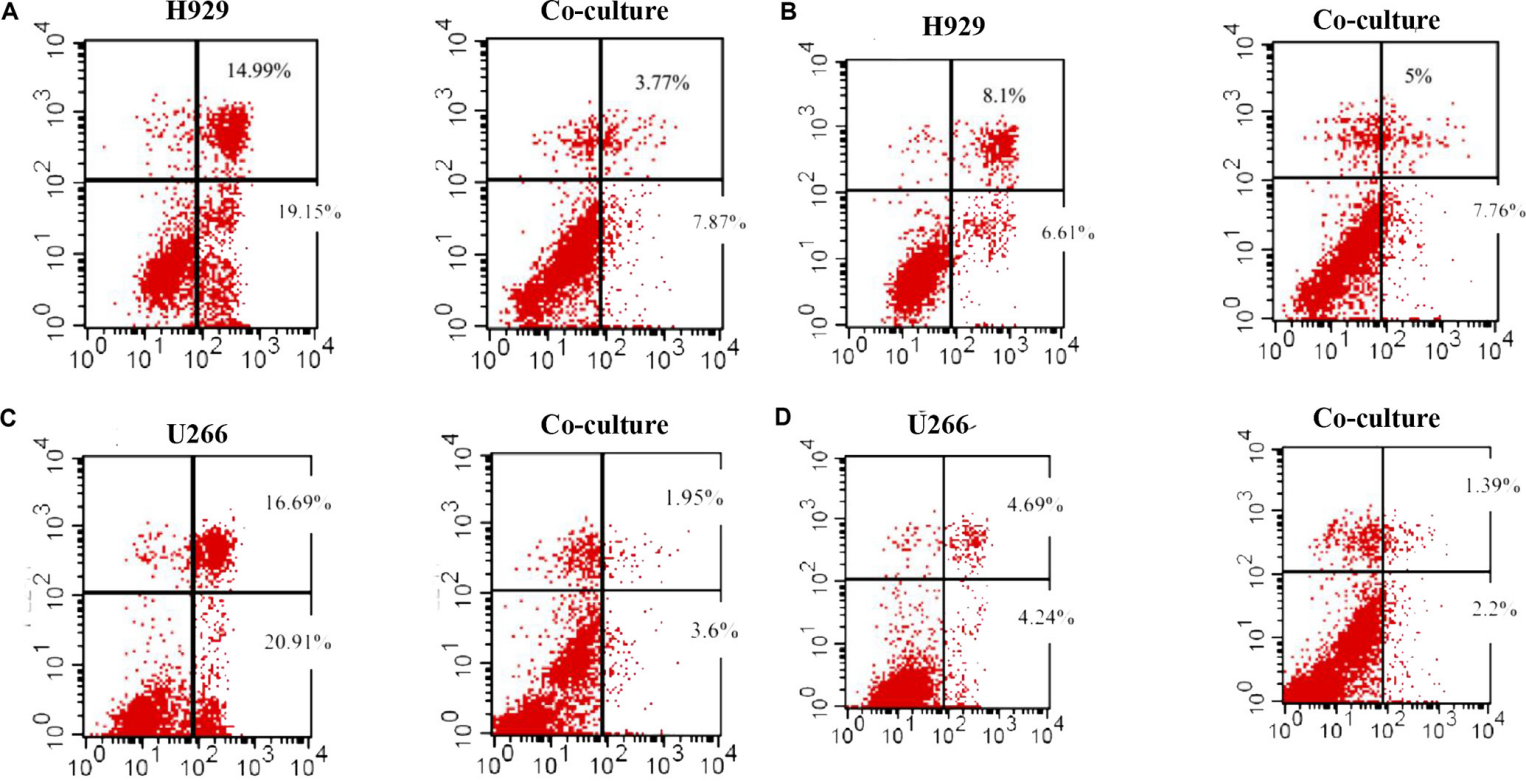
Adipocytes protects MM cells from apoptosis when cultured together The apoptosis of MM cells is detected by Flow cytometry. The percentages of apoptotic cells are reduced in co-culture condition when adding bortezomib and dexamethasone. (**A**) bortezomib treatment in H929 cells; (**B**) dexamethasone treatment in H929 cells; (**C**) bortezomib treatment in U266 cells; (**D**) dexamethasone treatment in U266 cells)

The above data suggested that the proliferation of myeloma could be affected by leptin, so we wondered whether the anti-tumor effect of chemotherapy could be influenced by adipocytes secreted leptin. Bortezomib and dexamethasone, two common chemotherapeutic agents, were added into U266 and NCI-H929 cells. The IC50 values were calculated based on the CCK-8 tests (Table 5). When co-cultured with adipocytes, the tumor-killing effect of 7nM bortezomib was decreased in U266 cells, as well as 30uM dexamethasone. Similar results were observed in H929 cells (Figure 4).

**Table 5:** IC50 values of Bortezomib and dexamthasone in U266 and NCI-H929

Cell line	Bortezomib	Dexamthasone
U266	7 nM	30 μM
NCI-H929	3 nM	25 μM

### Adipocytes increase the expression of proliferation and anti-apoptosis proteins

**Figure 5:**
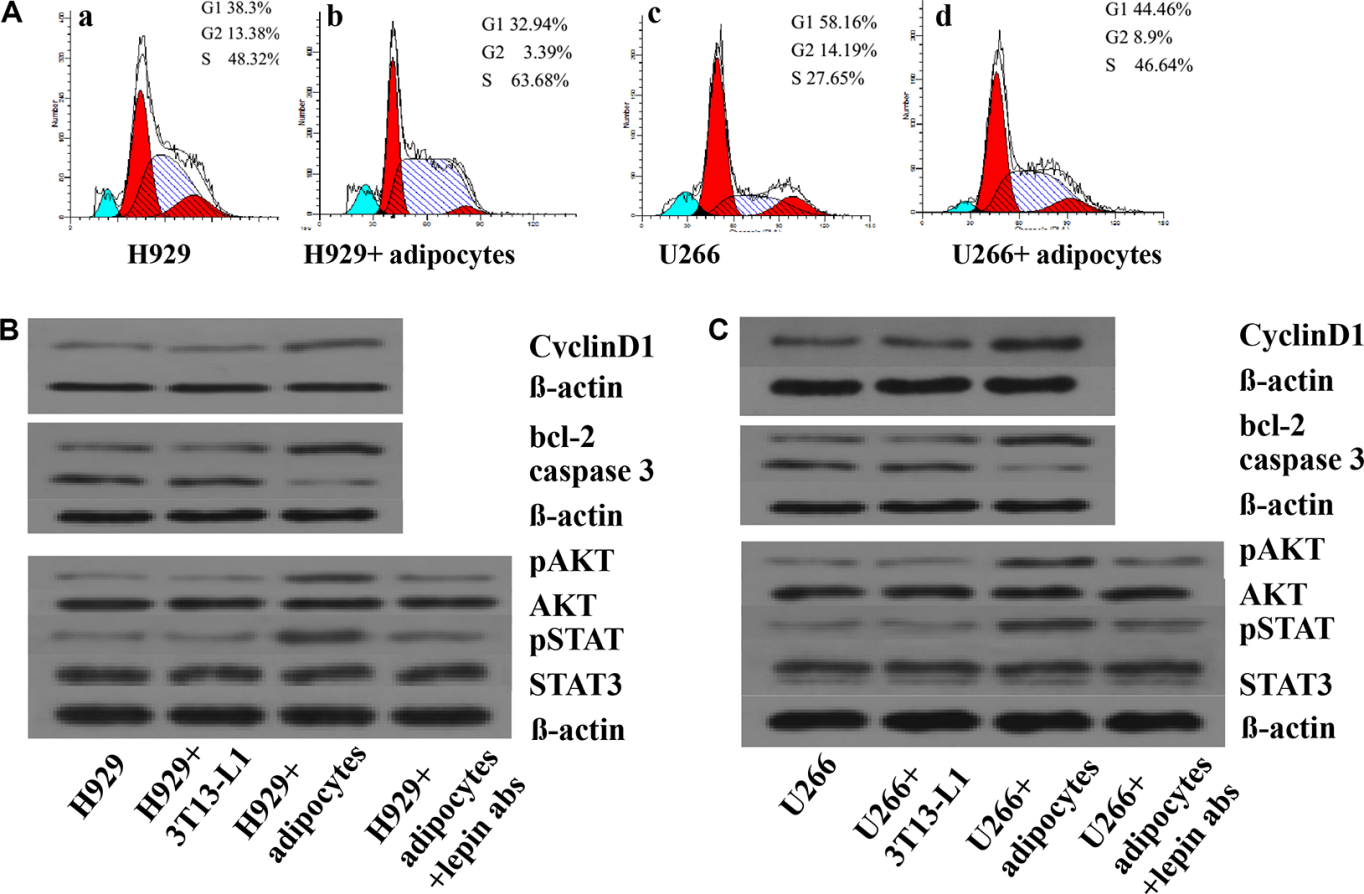
Adipocytes influence the cell cycle and protein expressions in MM cells The percentages of MM cells in different phases of cell cycle are measured by Folw cytometry. In Figure 5A-a and 5A-c, the MM cells are served as controls. In Figure 5A-b and 5A- d, the MM cells are co-cultured with adipocytes. The percentages of cells in S phase are increased in 5A-b and 5A-d. Next, the expression of proteins including CyclinD1, bcl-2, caspase 3, pAKT, AKT, pSTAT, STAT3, and beta-actin are detected by Western blotting.(**A**) percentages of cells in S phase increased in co-culture system; (**B**) levels of proliferation associated proteins up-regulated in H929 cells; (**C**) levels of proliferation or apoptosis associated proteins up-regulated in U266 cells. When adding leptin antibodies to the co-culture system, the levels of proliferation associated proteins decreased.

To explore the reasons for increasing proliferation and inhibiting apoptosis when myeloma cells were co-cultured with adipotcytes, the expressions of cell cycle, proliferation and apoptosis related proteins were measured. The data showed that the percentage of U266 cells in G1 phase decreased from 58.16% to 44.46%, along with S phase increased from 27.65% to 46.64%. The same trend was also found in H929 cells with the G1 phase declined from 38.3% to 32.9%, along with S phase elevated from 48.32% to 63.38%. The expression of cyclinD1, phosphorylated AKT and STAT3 proteins in myeloma cells were increased when co-cultured with adipocytes, whereas the percentage of phosphorylated proteins decreased when using anti-lepin receptor antibody(Figure 5). We also tested the changes of apoptosis related proteins in co-culture system, and found increased expression of Bcl-2 and decreased expression of caspase-3 (Figure 5).

### Leptin promotes proliferation of multiple myeloma cells trough activating JAK/STAT-PI3K/AKT pathway

**Figure 6:**
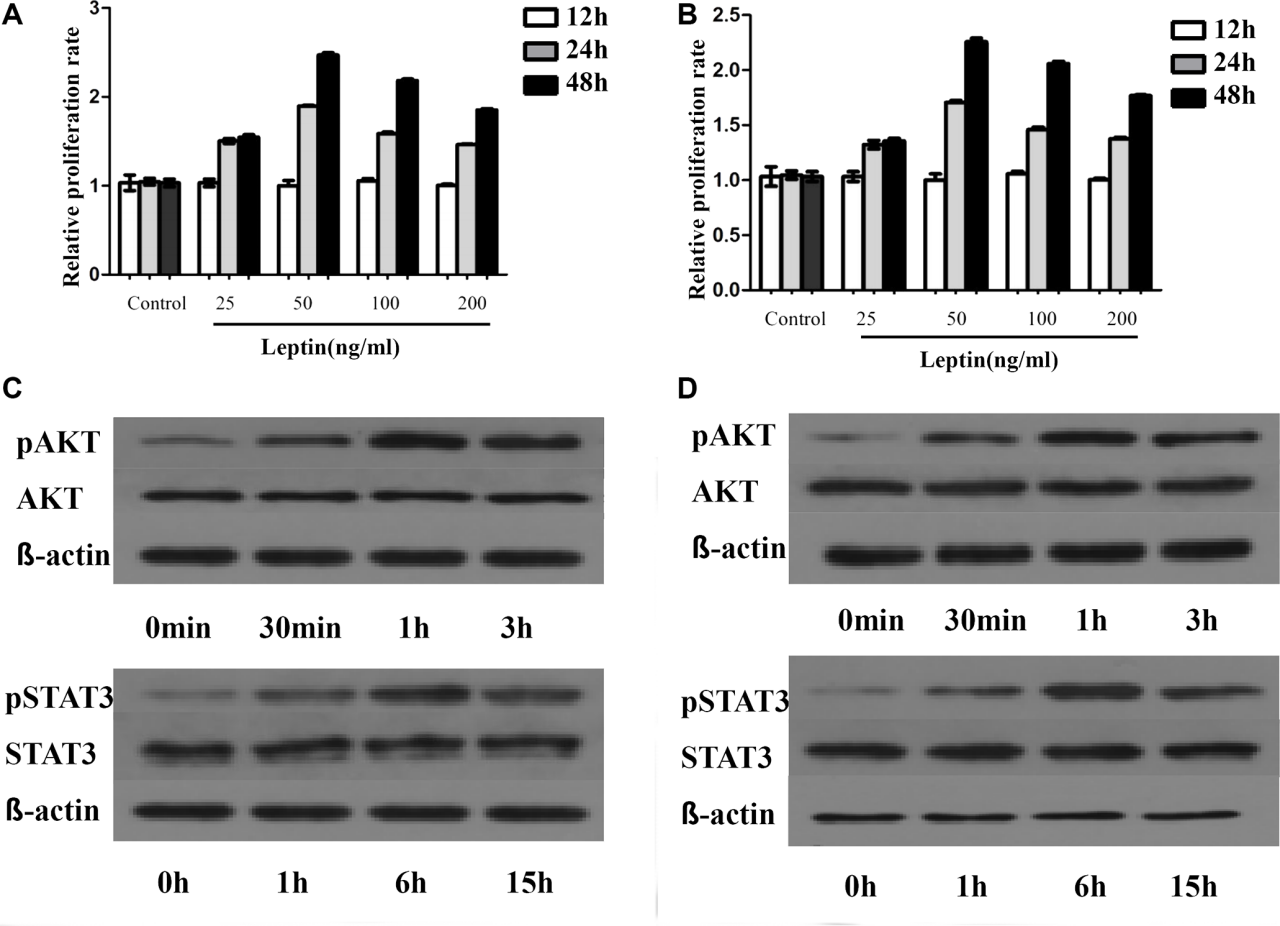
Leptin promotes MM cells proliferation by regulating phosphorylation of proliferation associated proteins The proliferation of MM cells is dependent on dose but not time. The most significant increase in proliferation is observed at 50ng/ml of leptin. This dose of leptin is used for the following experiments. (**A**) proliferation rates of H929 at different times with various concentrations of leptin; (**B**) proliferation rates of U266 at different times with varied concentrations of leptin; (**C**) levels of phosphorylated AKT and STAT3 at different time points in H929 cells treated with 50 ng/ml leptin; (**D**) levels of phosphorylated AKT and STAT3 at different time points in U266 cells treated with 50 ng/ml leptin)

**Figure 7:**
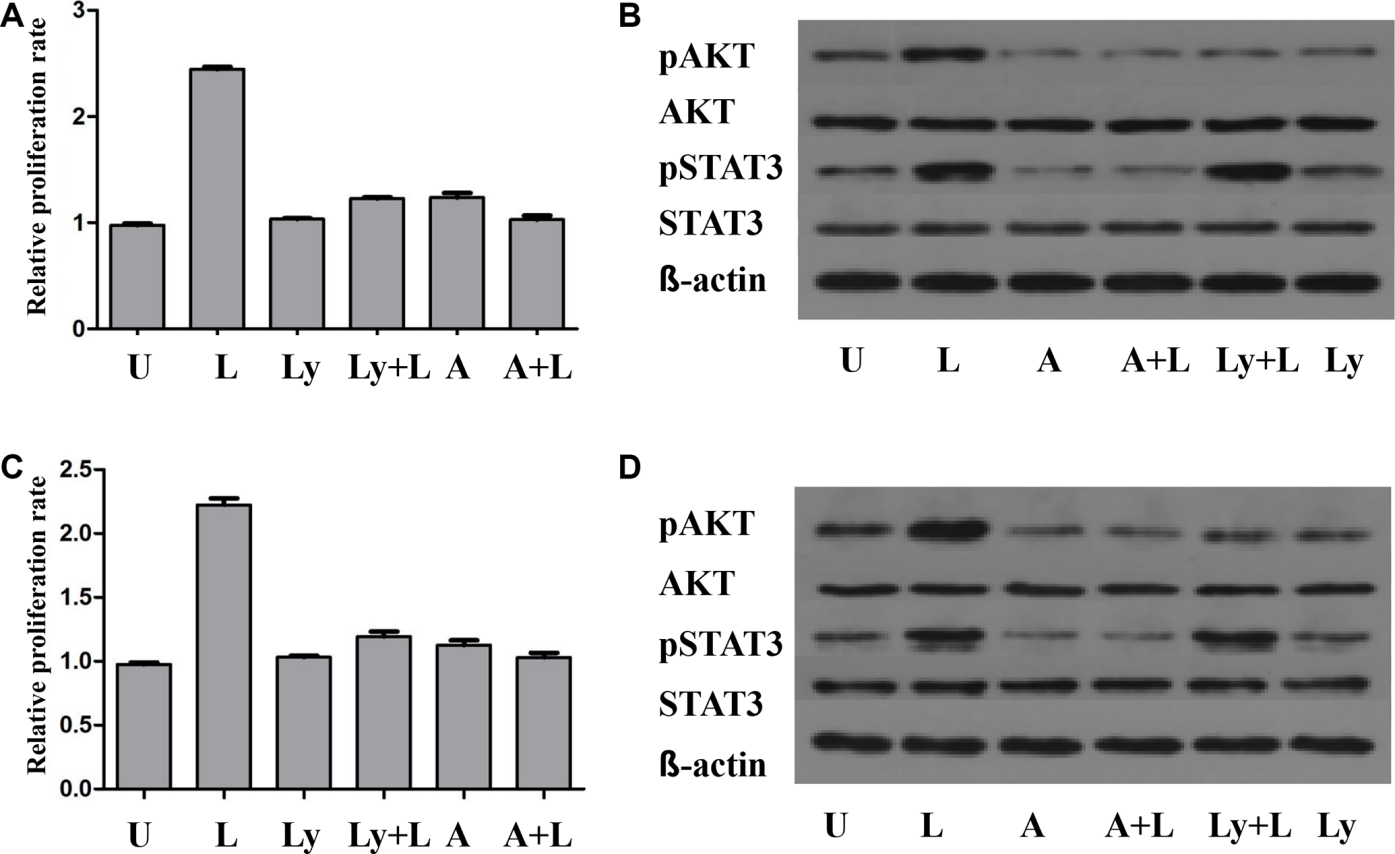
The pro-proliferation effect of leptin is influenced by the JAK/STAT-PI3K/AKT pathway The proliferation of MM cells is significantly increased when treated with leptin. This effect of leptin is suppressed by either LY294002 or AG490, along with decreased level of pAKT. The levels of pSTAT3 are reduced in the presence of AG490, but not LY294002. (**A**) proliferation rates of H929 cells under different treatments; (**B**) levels of phosphorylated AKT and STAT3 in H929 cells under different treatments; (**C**) proliferation rates of U266 cells under different treatments; (**D**) levels of phosphorylated AKT and STAT3 in U266 cells under different treatments. p, phosphorylated; U, untreated; L, leptin; A, JAK/STAT inhibitor AG490; LY, PI3K inhibitor LY294002).

As illustrated in Figure 6, leptin treatment stimulated the growth of U266 and 929 cells in a time-dependent manner, but not a dose-dependent manner. Leptin stimulated the growth of both cells most at the concentration of 50ng/ml. AKT phosphorylation was increased to peak at 1h after treatment, while the AKT protein expression was not changed. STAT3 phosphorylation was increased to peak at 6h of treatment and the increase was not due to the increased STAT3 protein expression. LY294002 specifically inhibited the phosphorylation of AKT, without affecting the expression of AKT or level of phosphorylated STAT3. Treatment with AG490, inhibitor of JAK/STAT, blocked leptin-induced hyperphosphorylation of both ERK and AKT. Simultaneous treatment with leptin and AG490 could not restore the level of phosphorylation of STAT3 and AKT. These data suggest that activation of JAK/STAT is upstream of the activation of AKT pathway. Both AG490 and LY2904002 inhibited leptin-induced proliferation of MM cells (Figure 7).

## DISCUSSION

The current study proposed that adipokines were dys-regulated and participant in cancer development and chemo-resistance focusing on MM. We have identified that the level of leptin is increased and adiponectin is decreased in newly diagnosed myeloma compared to healthy controls. When co-culturing with adipocytes, the proliferation of myeloma cells was increased and the anti-tumor efficacy of chemotherapy drugs was reduced, associated with up-regulation of cell cycle and anti-apoptosis proteins via leptin. Furthermore, we found that JAK/STAT-PI3K/AKT pathway should be responsible for the pro-tumor effects of leptin. These findings are in agreement with the results obtained by other researchers [16, 23].

Elevated level of leptin can be a factor for fostering development and progression of tumors as it has the characteristics of mitogenic, proinflammatory, antiapoptotic, and proangiogenic [16, 24]. Few studies [11, 17, 24–26] have investigated the effects of leptin in hematological tumors and found that leptin modestly promotes proliferation of myeloma cell lines, and reduces chemotherapy induced apoptosis in myeloma cell lines, which are in accordance with our results. In addition, Leptin was correlated with ISS stage in patients with multiple myeloma. Moschovi et al. [27] showed that leptin levels were higher at diagnosis of multiple myeloma when compared with controls.

A prospective study revealed that low level of adiponectin was associated with high risk of colon cancer [28] and breast cancer [29]. We also found a decreased expression of adiponectin in newly diagnosed multiple myeloma patients compared to healthy controls. And its level further decreased in patients with advanced stage of disease. Adiponetin level was inversely correlated with IgG level, ß2M and ER. A possible explanation could be due to a possible protective effect of adiponectin against myeloma. These results were similar with the findings of Dalamaga et. al. [17] who also reported that low level of adiponectin could be a risk factor of multiple myeloma.

We used 3T3-L1 differentiated mature adipocytes to study the impact of leptin on the biological behaviors of multiple myeloma cells. We found that mature adipocytes could directly stimulate proliferation, accelerate cell cycle, and up-regulate cell cycle related proteins in multiple myeloma cells when cultured together. Several studies [17, 25, 26] have revealed that adipocytes secreted leptin could help tumor cells to proliferate, chemo-resistant, and metastasis via activating pro-tumor pathways. After adding anti-leptin related receptor antibodies, the proliferation of myeloma cells was inhibited, indicating leptin and its receptors were responsible for the pro-tumor effect of adipocytes. A few studies showed that adipocytes could protect myeloma cells from chemotherapy induced apoptosis by increasing expression of Bcl-2 and Pim-2 protein [30]. This was in accordance with our results that co-culture with adipocytes could reduce apoptosis by increasing Bcl-2 expression and inhibiting activation of caspase-3.

In order to well understand how leptin exert its role in promoting cancer, we treated myeloma cells with recombinant leptin at several concentrations and times. The results exhibited that leptin treatment stimulated the growth of U266 and H929 cells in a time-dependent manner, but not a dose-dependent manner. Leptin stimulated the growth of both myeloma cells via increasing AKT and STAT3 phosphorylation, without changing expression of AKT and STAT3. LY294002 specifically inhibited the phosphorylation of AKT, without affecting the expression of AKT or level of phosphorylated STAT3. Treatment with AG490 blocked leptin-induced hyperphosphorylation of both ERK and AKT. Simultaneous treatment with leptin and AG490 could not restore the level of phosphorylation of STAT3 and AKT. These data suggest that leptin stimulated MM cell growth through activating JAK/STAT-PI3K/AKT pathway.

## MATERIALS AND METHODS

### Study patients

A total of 28 patients with newly diagnosed disease were included from hematology department, Union hospital. The serum concentrations of adipokines including leptin, adiponectin, resistin, and visfatin were compared to those measured in 28 age- and sex- matched healthy volunteers. Plasma samples were taken immediately after diagnosis. The diagnostic criteria, clinical characteristics of myeloma patients have been detailed previously [1, 31]. All subjects provided informed consent and the study protocol was approved by the Ethics Committee of the Tongji Medical college, Huazhong University of Science & Technology.

### Determination of serum and supernatants adipokine concentrations

The plasma levels of leptin, adiponectin, resistin, and visfatin were detected by the enzyme-linked immunosorbent assay (ELISA) kit(Elabscience, Inc.). The supernatants collected from cell culture media of myeloma or adipocytes in serum-free medium for 12, 24, 48 hours. The concentration of leptin in conditioned media were tested by ELISA kit(Elabscience, Inc.). All measurements were performed according to the instructions of the manufactures.

### Cell culture

RPMI8226, ARH-77, U266, NCI-H929 and 3T3-L1 cells were purchased from American Type Culture Collection (ATCC). Primary MSCs were isolated from BM aspirates of healthy control volunteers using anti-CD138 antibody coated magnetic beads (Miltenyi Biotec, Inc.). Cells were cultured in conditioned medium with 10% fetal bovine serum (FBS) according to the ATCC cell culture suggestion. Multiple myeloma cells were grown in RPMI-medium containing 2 mmol/L glutamine, 20% FCS, 100 U/mL penicillin and 0.1 mg/mL streptomycin.

### Generation of mature adipocytes *in vitro*

As previously reported, the MSCs were prepared using the BM cells from human fetal femoral bones[32]. MSCs were identified by the flow cytometry, and cultured in adipocyte medium for 2 weeks to get mature adipocytes. The adipocyte precursor (pre-adipocyte) cell line 3T3-L1 cells were also used to generate mature adipocytes. After differentiation, these cells were fixed with 4.0% paraformaldehyde, stained with red oil and observed using microscopy as previously reported [33].

### Co-cultures of MM cells and mature adipocytes

3T3-L1 cells were seeded at the bottom of 6 well plates, and induced to mature adipocytes as mentioned above. Abandon culture medium when the differentiation rate was among 80% to 90%, and washed three times gently with PBS. The multiple myeloma RPMI8226, ARH77, U266, and H929 cells were adjusted to the appropriate density using serum-free RPMI1640 medium. MM cell suspension was added to the mature adipocytes, and myeloma cells without adipocytes were used as control group. In some experiments dexamethasone or bortezomib was added for 12, 24 and 48 hours.

### Proliferation assay by CCK-8

Myeloma cells(1×10^5^/ml) were seeded in 96 culture wells for different interventions. After 12, 24, and 48 hours of culture, the proliferation assay was performed using CCK-8, according to the manufacturer’s insrtuctions.

### Apoptosis assay

After treating cells with specific agents for 12, 24, and 48 hours, an annexin V-binding assay was performed to measure proportion of cell apoptosis by using a BD flow cytometer, accorinding to the manufacturer’s insrtuctions.

### Western blot analysis

After leptin or chemotherapy treatment, multiple myeloma cells were collected and lysised for the following experiments. Briefly, sodium dodecyl sulfate-polyacrylamide gel electrophoresis (SDS-PAGE) system was used to separate proteins from cell lysates. Cell proteins were next transferred to a nitrocellulose membrane, and immunoblotted with antibodies against CyclinD1, caspase 3, Bcl-2 and phosphorylated (p) or non-phosphorylated STAT3 and AKT. β-actin protein levels were used to serve as a loading control.

### Statistical analysis

Statistical analysis was carried out using SPSS 14.0 for Windows software. Variables were expressed as mean ± standard deviation (SD). Differences between groups were analyzed by an unpaired *t* test. The correlation coefficients between two variable parameters were determined by Pearson correlation test. Significance was assigned for *P* values as significant where *P* < 0.05.

## CONCLUSIONS

In conclusion, elevated leptin level was found in newly diagnosed MM. Our study demonstrate that up-regulated leptin could stimulate proliferation and reduce the anti-tumor effect of chemotherapy in multiple myeloma possibly via activating AKT and STAT3 pathways, and leptin might be one of the potential therapeutic targets for treating myeloma.
