# Correction: Adipocytes secreted leptin is a pro-tumor factor for survival of multiple myeloma under chemotherapy

**DOI:** 10.18632/oncotarget.28236

**Published:** 2023-09-22

**Authors:** Wen Yu, De-Dong Cao, Qiu-bai Li, Hui-ling Mei, Yu Hu, Tao Guo

**Affiliations:** ^1^Department of Hematology, Union Hospital, Tongji Medical College, Huazhong University of Science and Technology, Wuhan, Hubei, China; ^2^Department of Pediatrics, Tongji Hospital, Tongji Medical College, Huazhong University of Science and Technology, Wuhan, China; ^3^Department of Oncology, Remmin Hospital of Wuhan University, Wuhan, Hubei, China; ^4^Collaborative Innovation Center of Hematology, Huazhong University of Science and Technology, Wuhan, Hubei, China; ^*^These authors contributed equally to this work

**This article has been corrected:** In Figure 5B, the western blots for bcl-2, caspase 3 and β-actin are accidental duplicates of those in Figure 5C. In addition, in Figure 6, the same Y-axis scale has now been applied for both Figure 6A and 6B, in order to better show the proliferation differences between the two cells. The corrected Figures 5 and 6, produced using the original data, are shown below. The authors declare that these corrections do not change the results or conclusions of this paper.

Original article: Oncotarget. 2016; 7:86075–86086. 86075-86086. https://doi.org/10.18632/oncotarget.13342

**Figure 5:**
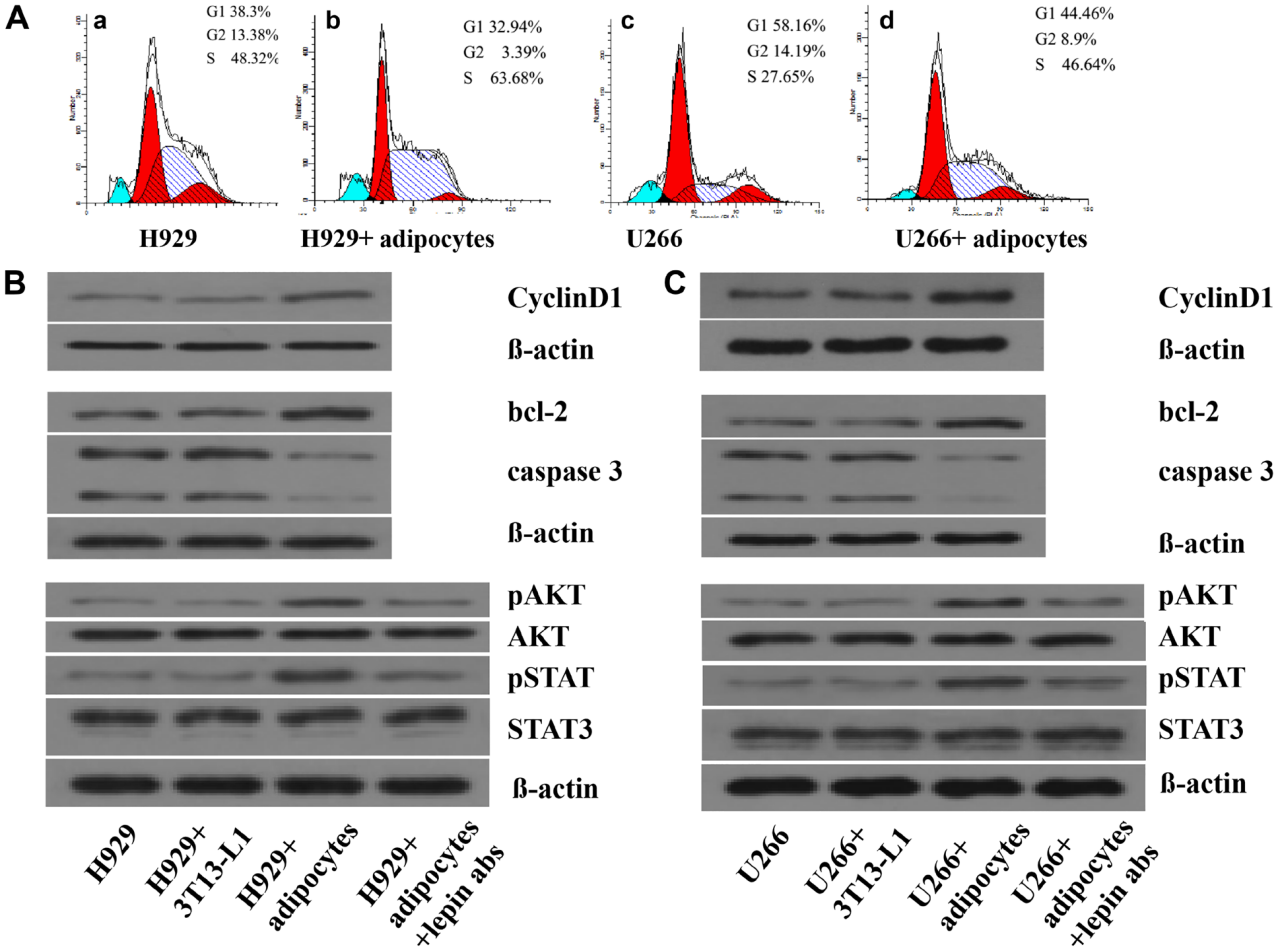
Adipocytes influence the cell cycle and protein expressions in MM cells. The percentages of MM cells in different phases of cell cycle are measured by Folw cytometry. In Figure 5A-a and 5A-c, the MM cells are served as controls. In Figure 5A-b and 5A-d, the MM cells are co-cultured with adipocytes. The percentages of cells in S phase are increased in 5A-b and 5A-d. Next, the expression of proteins including CyclinD1, bcl-2, caspase 3, pAKT, AKT, pSTAT, STAT3, and beta-actin are detected by Western blotting. (**A**) percentages of cells in S phase increased in co-culture system; (**B**) levels of proliferation associated proteins up-regulated in H929 cells; (**C**) levels of proliferation or apoptosis associated proteins up-regulated in U266 cells. When adding leptin antibodies to the co-culture system, the levels of proliferation associated proteins decreased.

**Figure 6:**
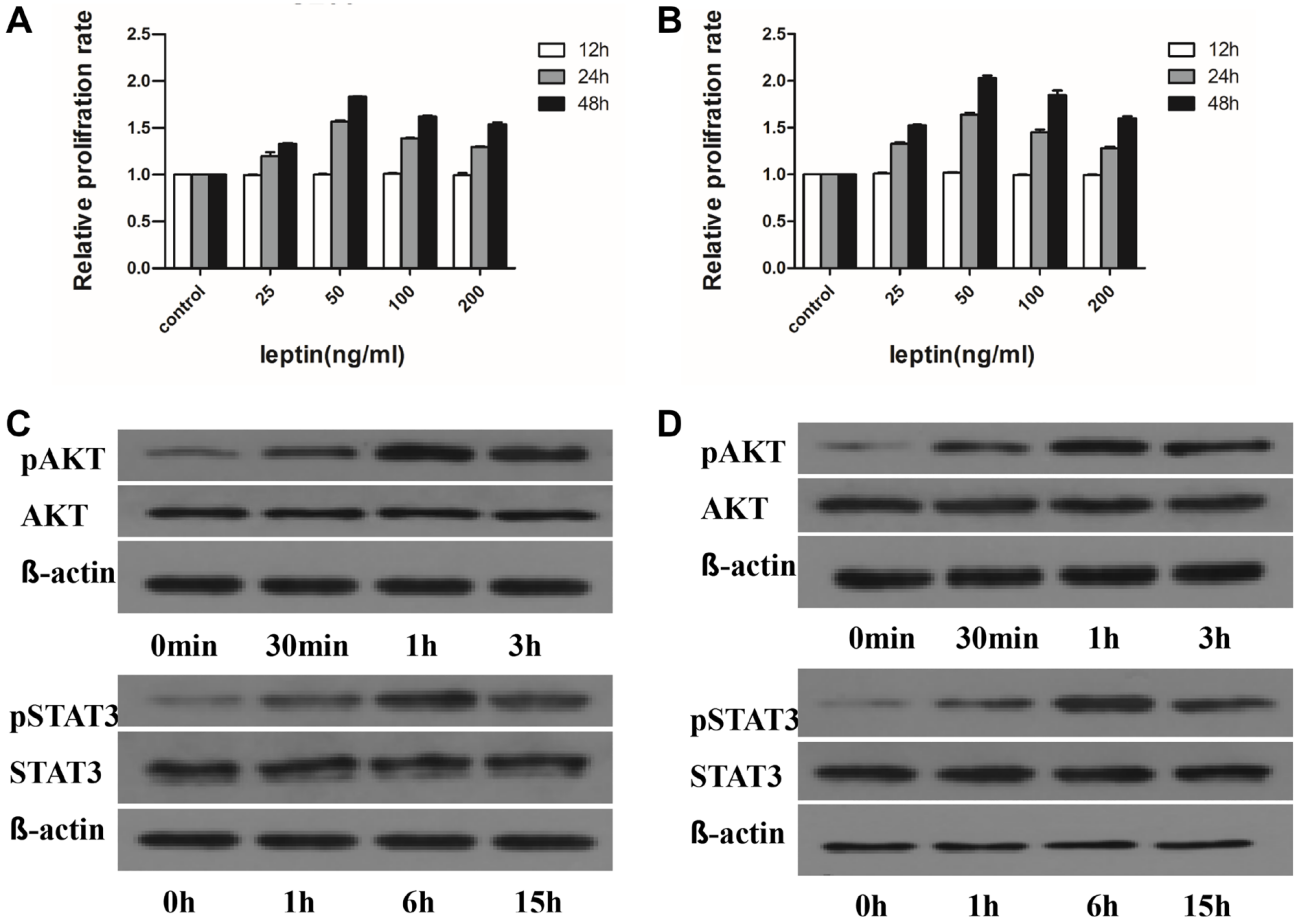
Leptin promotes MM cells proliferation by regulating phosphorylation of proliferation associated proteins. The proliferation of MM cells is dependent on dose but not time. The most significant increase in proliferation is observed at 50 ng/ml of leptin. This dose of leptin is used for the following experiments. (**A**) proliferation rates of H929 at different times with various concentrations of leptin; (**B**) proliferation rates of U266 at different times with varied concentrations of leptin; (**C**) levels of phosphorylated AKT and STAT3 at different time points in H929 cells treated with 50 ng/ml leptin; (D) levels of phosphorylated AKT and STAT3 at different time points in U266 cells treated with 50 ng/ml leptin).

